# Investigation of the cytotoxicity induced by didocosahexaenoin, an omega 3 derivative, in human prostate carcinoma cell lines

**DOI:** 10.1016/j.crphar.2022.100085

**Published:** 2022-01-19

**Authors:** Glenn F. Robinson, Kartheek KY. Sooda, Roger M. Phillips, Simon J. Allison, Farideh A. Javid

**Affiliations:** Department of Pharmacy, School of Applied Sciences, University of Huddersfield, Queensgate, Huddersfield, HD1 3DH, UK

**Keywords:** Didocosahexaenoin, Omega-3 fatty acids, Prostate cancer, ROS, Caspase 3/7

## Abstract

The aim of the present study was to investigate the cytotoxicity induced by an omega-3 derivative, didocosahexaenoin (Dido) on human prostate carcinoma cells and to compare the cytotoxicity to that of docosahexaenoic acid (DHA). Different carcinoma- and non-carcinoma cells were exposed to various concentrations of omega-3 compounds at varying exposure times and the cytotoxicity was measured by MTT assay. The mechanism of Dido-induced apoptosis was investigated in prostate carcinoma cells. Dido induced stronger cytotoxicity than DHA in human prostate carcinoma cells in a dose- and time-dependent manner. Dido was also more selective and potent in inducing cytotoxicity in prostate carcinoma cells than other carcinoma cell lines tested. Pre-treatment with Dido increased the level of reactive oxygen species (ROS) in prostate carcinoma cells. Pre-treatment with various antioxidants reduced the cytotoxicity induced by Dido. Pre-treatment with Dido ≥30 ​μM also induced apoptosis which was suggested to involve an externalisation of phosphatidyl serine, a significant increase in the mitochondrial membrane potential (p ​< ​0.01) and the level of activated caspase 3/7 (p ​< ​0.05) in prostate carcinoma cells. This study is the first to show that Dido induced cytotoxicity with high selectivity and higher potency than DHA in human prostate carcinoma cells. The mechanism of action is likely to involve an increase in the level of ROS, loss in the mitochondrial membrane potential as well as externalisation of phosphatidyl serine and increase in the caspase 3/7 activity. Dido may have potential to be used for the adjuvant therapy or combination therapy with conventional chemotherapeutic drugs.

## Highlights

• Didocosahexaenoin, an omega-3 derivative, induces cytotoxicity in prostate carcinoma cells.
• The cytotoxicity induced by didocosahexaenoin in carcinoma cells was more potent than that afforded by DHA.
• The likely mechanisms involve an increase in the level of ROS, caspase 3/7 activity, and loss of MMP.

## Graphical abstract

**Figure:**
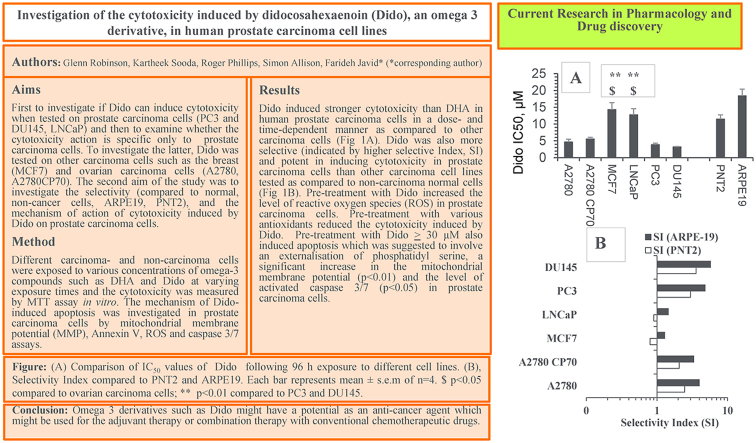

## 1 Introduction

Prostate cancer is the fifth leading cause of death worldwide and the second most commonly diagnosed cancer in men, accounting for 15% of all new cancer cases worldwide and 7% of male cancer deaths (Rawla, 2019; Ferlay et al., 2015). Current treatment options include surgery, radiotherapy, hormone therapy or ablation, which includes cryotherapy and high-intensity ultrasound. In cancer patients who no longer respond to hormone therapy, chemotherapy is a valuable option (Cancer Research UK, 2020). Current drugs used clinically to treat prostate cancer include docetaxel and mitoxantrone. Docetaxel is a mitotic spindle poison which inhibits microtubule depolymerisation, resulting in mitotic cell cycle arrest (Fabbri et al., 2008). Mitoxantrone is a topoisomerase II inhibitor and causes the accumulation of cellular DNA damage resulting in cellular demise by apoptosis (Zhao et al. 2011). Although chemotherapeutic drugs for certain cancer indications significantly improve patient survival, for many of the current therapies, treatment effectiveness is severely restricted by dose limiting toxicities, serious side effects include long-term heart failure and/or damage to reproductive organs (Fenton et al., 2018; Meirow and Nugent, 2001; Kremer et al., 2001). Additionally, more immediate side effects such as nausea and vomiting, hair loss, fatigue and the emotional toll can encourage patients to discontinue their treatment (Litwin and Tan, 2017). Thus, there is an unmet clinical need for developing novel therapies with minimum side effects.

Epidemiological studies have shown that changes in migration and food consumption patterns have had a significant effect on the incidence of cancers (Mohammad et al., 2009; Gerber, 2012) and many studies have shown that a diet rich in oily fish (containing omega-3 oil) has been correlated with a lower occurrence of cancer in the population (D'Angelo et al., 2020; Larsson et al., 2004; Wall et al., 2010; Terry et al., 2004; Gerber, 2012). Epidemiological studies, however, have not unequivocally proved that fish consumption is associated with a lower incidence of cancer, although there is substantial evidence to indicate omega-3 fatty acids inhibit tumourigenesis (Larsson et al., 2004).

There is substantial literature to suggest that the omega-3 polyunsaturated fatty acids docosahexaenoic acid (DHA) and eicosapentaenoic acid (EPA) have not only anti-inflammatory activity (Wall et al., 2010; Serhan et al., 2004; Calder, 2013) but also anti-tumour properties (D'Angelo et al., 2020; Seiler et al., 2018; Aucoin et al., 2017; Gu et al., 2013; Brown et al., 2013; Laviano et al., 2013; Gerber, 2012). EPA and DHA can be synthesised in the body from alpha-linolenic acid found commonly in vegetable oils, however, the conversion rate is very low (Hull, 2011; Berquin et al., 2011). Therefore, DHA and EPA must be supplemented in the diet if they are to be present in the body in high concentrations. DHA is known to have effects on membrane structure and function and can alter the effects of resident proteins such as Raf-1 kinase, tyrosine kinases and ion channels (Mohammad et al., 2009). Both EPA and DHA have also been shown to be involved in programmed cell death, autophagy and ROS production contributing to their anti-tumour activity (Fukui et al., 2013; Jing et al., 2011; Kang et al., 2010; Shin et al., 2013). Evidence suggests that DHA as a free fatty acid induces apoptosis in MCF-7 ​cells, human breast cancer cells, through the selective activation of caspase 8 (Seiler et al., 2018; Kang et al., 2010).

DHA has also been shown to induce autophagy in tandem with apoptosis as confirmed by the presence of LC3-II (Shin et al., 2013). Excessive levels of Reactive Oxygen Species (ROS) can cause irreparable damage to cells resulting in induction of autophagy or apoptosis. ROS also regulate transcription factors, gene expression, differentiation and proliferation and has been seen in elevated levels when prostate cancer cell lines were treated with DHA (Jing et al., 2013; Shin et al., 2013). DHA has also been found to improve the efficacy of anti-tumour agents in pre-clinical studies as an adjuvant with no side effects (Siddiqui et al., 2011). In particular, DHA was found to improve the uptake of anti-tumour agents by resistant cells. Interestingly, a study in 2014 indicated that doxorubicin-induced anti-tumour effects were enhanced by the addition of stearidonic acid (an 18-carbon omega-3 fatty acid) in PC3 and LNCaP prostate carcinoma cell lines (Trebelhorn et al., 2014). Therefore, previous studies have focused on the effect of omega-3 fatty acids, in particular DHA and EPA on prostate cancer progression. Such studies reported the activation of multiple mechanisms including inhibition of formation of prostaglandin E2 via cyclooxygenase 2 pathway (Lands, 1992), lipoxygenase activity, toll-like receptors, inhibition of NF-kB, activation of PPAR-gamma (Im, 2016; Kliewer et al., 1997), and formation of pro-resolvin metabolites (Serhan and Petasis, 2011). Furthermore, involvement of caspases and ROS were also reported (Shin et al., 2013; Kang et al., 2010; Jing et al., 2011).

**Fig. 1:**
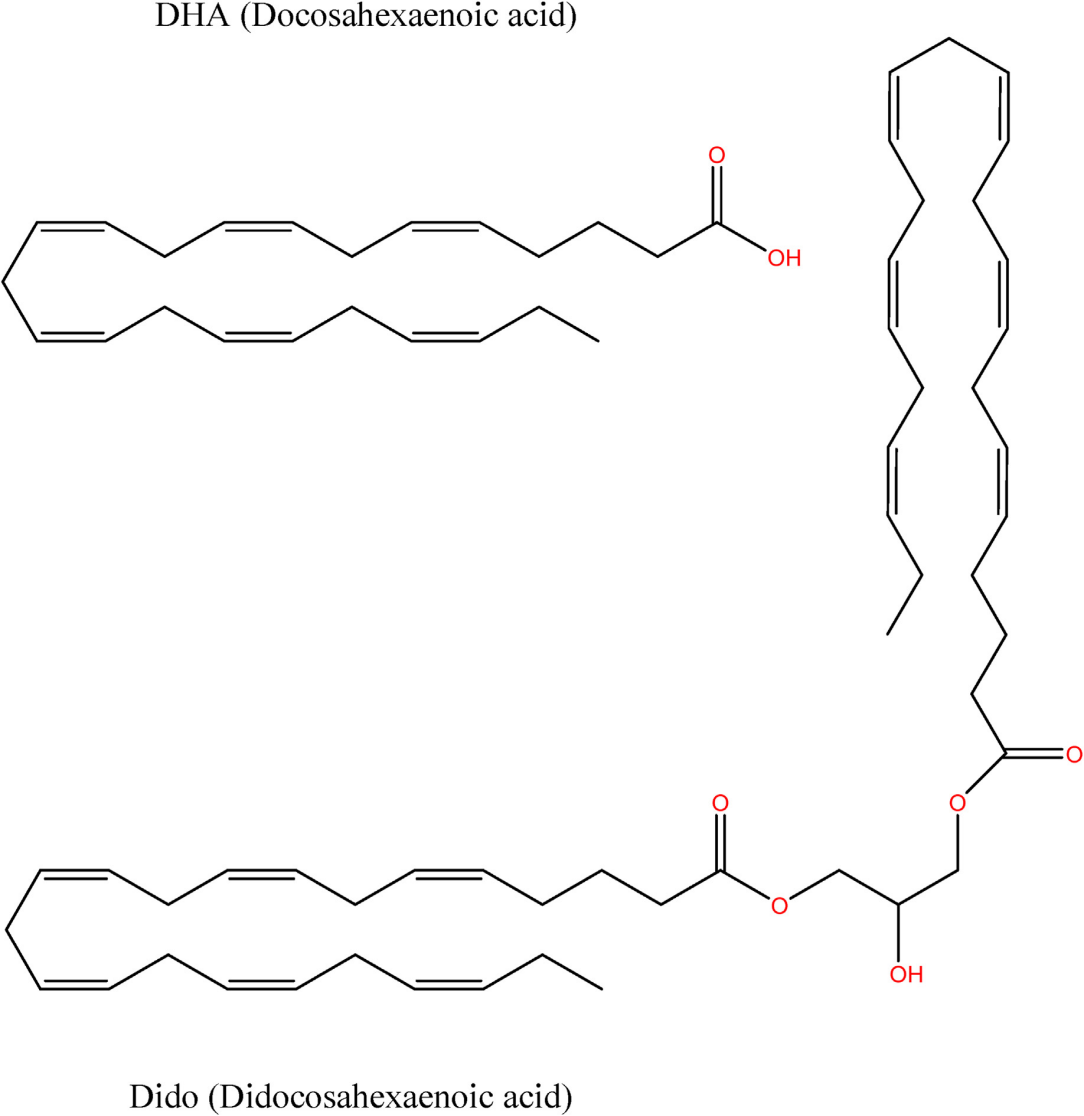
Dido can be synthesised from DHA. For the synthesis of 1,3-diglycerides, the free hydroxyl groups of commercially available glycerol 2-benzyl ether could be reacted with the preferred fatty acid derivative and a suitable coupling reagent. The benzyl group can be removed by hydrogenolysis. Glycerol might also be selectively 1,3-diacetylated in the presence of the preferred fatty acid derivative and a suitable coupling reagent.

Didocosahexaenoin (Dido) is a diglyceride of DHA and can be synthesised from DHA triglycerides using gastric and pancreatic diacylgycerol lipases (Fig. 1) (Currie et al., 2016). The lipases break down triglycerides into more polar lipids with increased water solubility which are able to form micelles (Reis et al., 2008). Medium chain triglycerides which contain fatty acids with a chain length of 6–12 carbon atoms such as lauric acid are rapidly hydrolysed by these pancreatic lipases and are transported to the liver whereas the omega-3 derivatives, particularly those at the sn-2 position (stereospecific number position of glycerol) such as 2-arachidonylglycerol (the endocannabinoid 2-AG), accumulate in cell membranes and are stored for future applications.

Whilst the free acid DHA has been the focus of a number of investigations, particularly in the potential treatment of cancer and in inflammation (Shin et al., 2013; Gleissman et al., 2010; Jing et al., 2011; Kang et al., 2010; Liu et al., 2016), no previous studies have reported any biological activity of its diglyceride derivative, Dido. Therefore, the aims of the present study were first to investigate if Dido can induce cytotoxicity when tested on prostate carcinoma cells and then to examine whether the cytotoxicity action is specific only to prostate carcinoma cells. To investigate the latter, we have also tested Dido on other carcinoma cells such as the breast and ovarian carcinoma cells. The second aim of the study was to investigate the selectivity (compared to normal, non-cancer cells), and the mechanism of action of cytotoxicity induced by Dido on prostate carcinoma cells.

## 2 Methods

### 2.1 Cells/reagents

PC-3 (human prostate adenocarcinoma cells), PNT2 (human normal prostate cells), MCF-7 (human mammary adenocarcinoma cells), A2780 (human ovarian carcinoma cells) and A2780-CP70 (human ovarian carcinoma cells, which have acquired *cis*-platin resistance) were maintained using RPMI 1640 Media supplemented with 10% (v/v) foetal bovine serum (FBS), 1% (v/v) penicillin streptomycin, 1% (v/v), L-glutamine and 1% (v/v) sodium pyruvate. DU145 (human prostate carcinoma cells) were maintained using EMEM with 10% (v/v) foetal bovine serum and 1% (v/v) penicillin streptomycin. ARPE19 (human normal retinal pigment cells) were maintained using 1:1 ratio DMEM F12 nutrient mixture: DMEM supplemented with 10% (v/v) foetal bovine serum, 1% (v/v) penicillin streptomycin, 1% (v/v), L-glutamine and 1% (v/v) sodium pyruvate. LNCaP (human prostate carcinoma cells) were maintained using 1:1 ratio DMEM:RPMI 1640 supplemented with 5% (v/v) foetal bovine serum, 1% (v/v) penicillin streptomycin, 1% (v/v), L-glutamine and 1% (v/v) sodium pyruvate. All cell lines were purchased from ATCC and grown under a humidified 5% CO_2_/95% air atmosphere at 37 ​°C, replacing the media every 2–3 days until approximately 70% confluency was achieved. All cell lines were used at passages lower than 20.

All reagents unless otherwise specified were purchased from Sigma Aldrich (Gillingham UK). Cell culture supplies and consumables were purchased from Sarstedt (Nümbrecht, Germany). Didocosahexaenoin (Dido) was purchased from Nu-chek prep (USA), DHA Ethyl Esters (EE) was purchased from BASF Pharma (Callanish, UK). Absolute ethanol was used to dissolve Dido. DHA was synthesised from ethyl docosahexaenoate (DHA EE) using previously described methods (Haraldsson et al., 2000). All fatty acids were dissolved in ethanol and the final concentration of ethanol did not exceed 1%. Preliminary experiments revealed that 1% ethanol did not induce any change in the cells as compared to media treated cells.

### 2.2 In *vitro* chemosensitivity assays

To determine whether Dido has any cytotoxic activity against cancer cells, chemosensitivity assays were performed on a panel of human carcinoma cells and non-carcinoma cells. Human prostate androgen-insensitive cancer cell lines PC3 (high metastatic potential) and DU145 (moderate metastatic potential), LNCaP (androgen sensitive prostate carcinoma cell), A2780 and A2780-CP70 (human ovarian carcinoma cells sensitive to and resistant to cisplatin, respectively), MCF-7 (breast carcinoma cells), PNT2 (non-carcinoma prostate cell line) and ARPE19 (retinal epithelial cells) were exposed to varying concentrations of Dido (1 ​nM–100 ​μM), or vehicle for 96 ​h contact time*. In vitro* chemosensitivity assays were performed using the MTT assay as previously described (Javid and Afshinjavid, 2015). At 70% confluency, cells were harvested by trypsinisation and seeded in 96-well cell culture plates at a density of 2000 ​cells/well. 24 h later, cells were exposed for 24, 48, 72 or 96 ​h to a range of concentrations of freshly prepared Dido.

In separate experiments human prostate carcinoma and non-carcinoma cells were used for the subsequent study of the pathways involved in Dido-induced cytotoxicity following an exposure to various compounds (1 ​nM–100 ​μM): Dido, DHA, ethyl docosahexaenoate (DHA EE), α-tocopherol (5 ​μM), ascorbic acid (250 ​μM), N-acetylcysteine (500 ​μM), or vehicle. All the effective concentrations were chosen from previous preliminary experiments. After the allocated contact time had elapsed, the media was removed and MTT (0.5% w/v) was added to each well. Following 4 ​h incubation time, the supernatants were removed, and formazan crystals dissolved in dimethylsulfoxide (150 ​μL) was added. The absorbance was read at 540 ​nm on a Tecan Infinite 50 UV plate reader.

### 2.3 Detection of ROS with 2′,7′-dichlorodihydrofluorescein diacetate (H2DCF-DA)

Cells were seeded at 6000 ​cells per well in black flat bottom Greiner CELLSTAR 96 well plates, (200 ​μL media per well) and incubated for 48 ​h. After 48 ​h, Dido (30 ​μM), α-tocopherol (5 ​μM), a combination of Dido (30 ​μM) plus α-tocopherol (5 ​μM), H_2_O_2_ (50 ​μM) or vehicle (1% v/v) was added to the wells and cells were further incubated for 1, 3 or 6 ​h under an atmosphere of 37 ​°C, 5% CO_2_/95% air. The media was then removed, and cells were washed with PBS 1x (200 ​μL, warmed to 37 ​°C) before a further 30 ​min incubation with 8 ​μM 2′,7′-dichlorodihydrofluorescein diacetate (H2DCF-DA). Cells were then washed with PBS to remove excess H2DCF-DA, and fluorescent intensity was read on an Optima FLUOstar microplate reader using a 492 ​nm emission filter and a 520 ​nm excitation filter.

### 2.4 Mitochondrial membrane potential measurement

In further experiments, attempts were made to measure the mitochondrial membrane potential (Δψ_m_) as a marker of early apoptosis (Ly et al., 2003). PC3 cells were seeded in 25 ​cm^2^ tissue culture flasks at a density of 37500 ​cells per flask. After 48 ​h incubation, Dido (10, 20, 30 or 40 ​μM) or ethanol (1% v/v) was added, and the treated cells were incubated for a further 6 or 24 ​h. After treatment, the cells were harvested by trypsinisation and washed with PBS 1x (5 ​mL) and centrifuged at 1200 ​rpm for 5 ​min. The supernatant was carefully aspirated off and the PBS wash step was repeated once more. The cells were counted on a NucleoCounter® NC-3000™ image cytometer and diluted to a final concentration of 1 ​× ​10^6^ ​cells/mL in PBS to which the dye, JC-1 was added at a final concentration of 2.5 ​μg ​mL^−1^. After 15 ​min incubation at 37 ​°C, cells were washed 2 times to remove excess JC-1 dye. The cells were centrifuged at 2800 ​rpm for 5 ​min at room temperature and the supernatant was carefully removed. The cell pellet was re-suspended, centrifuged and washed as per the manufacturer's protocol. The cell pellet was re-suspended in a solution of DAPI (1 ​μg ​mL^−1^ in PBS) and the effects on the mitochondrial membrane potential were quantified using the NucleoCounter® NC-3000™ system. This method allows identification of the levels of live, apoptotic, and dead cells. It has been done by measuring the mitochondrial transmembrane potential (Δψm), as its disruption is often linked to the early stages of apoptosis and the loss of it is associated with necrosis and apoptosis. The lipophilic cationic dye JC-1 (5, 5, 6, 6-tetrachloro-1, 1, 3, 3-tetraethylbenzimidazolcarbocyanine iodide) displays potential-dependent accumulation in the mitochondria. Healthy cells are recognized by JC-1 localization in the mitochondrial matrix due to negative charge formed by the intact mitochondrial membrane potential which induces red fluorescence. In cells that undergo apoptosis, mitochondrial potential collapses and JC-1 accumulates in the cytosol, establishing green fluorescence. Cells have also been stained with DAPI that recognizes necrotic and late apoptotic cells which appear in blue fluorescence, resulting in decreased red/green fluorescence intensity ratio.

### 2.5 Annexin V apoptotic assay

PC3 cells were seeded in 25 ​cm^2^ tissue culture flasks at a density of 180000 ​cells per flask. After 48 ​h, ethanol or Dido (10, 20, 30, 40 and 50 ​μM) was added. After a further 24 and 48 ​h contact time, all cells were collected (floating and adhered) and stained with annexin V-fluorescein isothiocyanate (FITC) and propidium iodide (PI, Roche) at room temperature for 15 ​min. Cells staining PI positive were considered either mechanically damaged or necrotic, cells staining with annexin V were considered early apoptotic and cells dual stained with both annexin V and PI suggests late apoptotic or necrotic cells. Cells not stained were considered to be live cells. The cells were then analysed on a FACSCalibur flow cytometer, where for each measurement 10000 events were counted.

### 2.6 Caspase 3/7 activation assay

PC3 cells were seeded at 8000 ​cells per well in black flat bottom Greiner CELLSTAR 96 well plates, (100 ​μL media per well) and incubated for 24 ​h at 37 ​°C under an atmosphere of 5% CO_2_/95% Air. After 24 ​h elapsed, the cells were exposed to Dido (10, 30 and 50 ​μM) or vehicle (1% v/v) and incubated for a further 6 ​h. Caspase 3/7 cleavage was then quantified using the ApoTox-Glo™ Triplex Assay kit (Promega) as per manufacturer's instructions.

### 2.7 Data analysis

Data analysis was expressed as mean ​± ​S.E.M, of minimum 4 separate experiments, n ​= ​4, unless otherwise indicated. The statistical significance was determined using IBM SPSS Statistics 22 using one way ANOVA with Games-Howell post hoc test or students t-test. P values of <0.05 were considered to be statistically significant.

The Cytotoxic Selectivity Ratio or Index was calculated (see Equation 1) in a modification of the hypoxic cytotoxic ratio and used as a means of indicating the selectivity of a drug between normal and cancer cell lines (Phillips et al., 1999).

$$CSR=\;\frac{normal\;cell\;IC_{50}\;of\;drug}{cancer\;cell\;IC_{50}\;of\;drug}$$

Equation1. Calculation of Cytotoxic Selectivity Ratio (CSR) as a representation of drug selectivity. A value ​> ​1 infers cytotoxic preference for cancer cells and a value ​< ​1 infers a cytotoxic preference for normal cells.

## 3 Results

### 3.1 *In vitro* cytotoxicity of Dido and other fatty acids on human prostate and other carcinoma and non-carcinoma cell lines

**Fig. 2:**
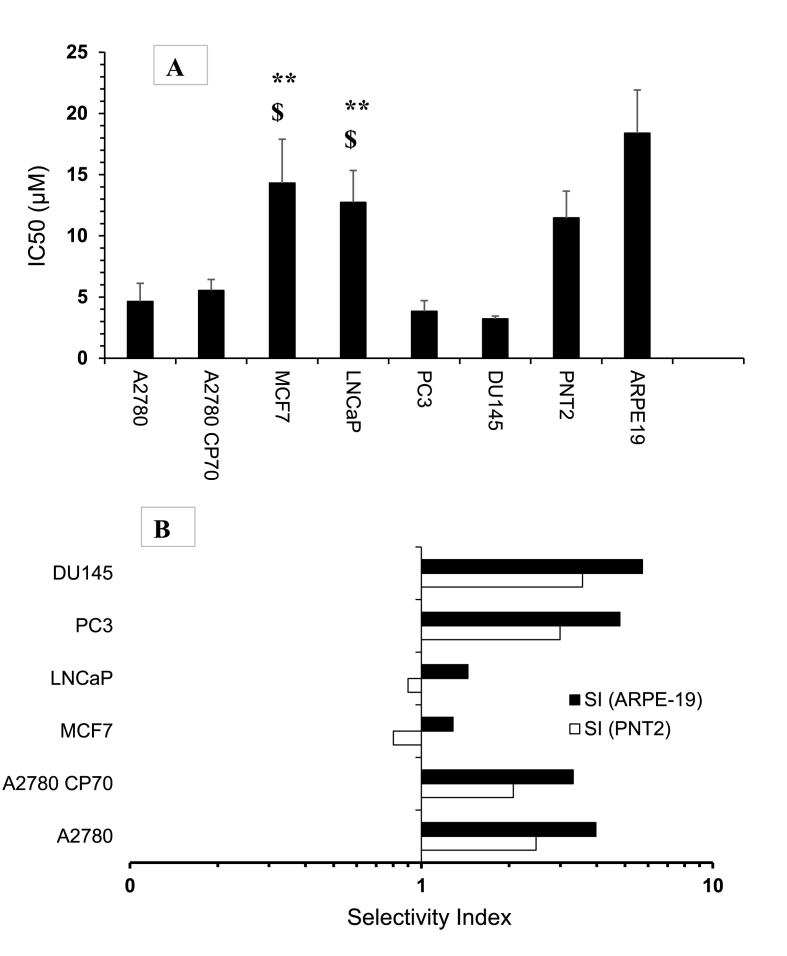
(A) Comparison of IC_50_ values of Dido following 96 ​h exposure to different cell lines. (B), Selectivity Index compared to PNT2 and ARPE19. PC3 (prostate adenocarcinoma cell line), A2780 (ovarian cancer cell line), A2780 CP70 (*cis*-platin resistant ovarian cancer cell line), ARPE19 (normal human retinal cells), DU145 (prostate cancer cell line), LNCaP (prostate cancer cell line), MCF7 (breast cancer cell line), PNT2 (human normal prostate cell line) Each bar represents mean ​± ​s.e.m of n ​= ​4. $ p ​< ​0.05 compared to ovarian carcinoma cells; ∗∗p ​< ​0.01 compared to PC3 and DU145.

Dido induced dose-dependent cytotoxicity with the strongest toxicity under 10.0 ​μM on DU145 and PC3 cells, A2780 and A2780-CP70 with IC50s of 3.20 ​± ​0.13 and 3.82 ​± ​0.50 ​μM, 4.6 ​± ​0.86, 5.53 ​± ​0.51 ​μM, respectively (Fig. 2A). However, Dido induced toxicity at higher concentrations than 10.0 ​μM in LNCaP and MCF-7 with significantly (p ​< ​0.01 and p ​< ​0.05) higher IC50s of 12.73 ​± ​1.84, 14.31 ​± ​2.07 ​μM, respectively as compared to prostate cell lines, PC3 and DU145, and ovarian carcinoma cells, A2780 and A2780-CP70 ​cells (Fig. 2A). The results show that Dido is less efficacious in inducing cytotoxicity in MCF-7 and LNCaP cell lines. Further experiments using non-carcinoma cell lines, ARPE119 and PNT2, showed that Dido induced cytotoxicity at much higher concentrations, with higher IC50 values of 18.38 ​± ​2.0 and 11.45 ​± ​1.26 ​μM, respectively as compared to those in prostate and ovarian carcinoma cells (Fig. 2A). The CSR values showed that Dido induced significant cytotoxicity with higher selectivity in PC3, DU145, A2780 and A2780-CP70 carcinoma cells compared to non-carcinoma cells, PNT2 and ARPE119 ​cells (Fig. 2B, Table 1).

**Table 1:** The Selectivity Index (Ratio) values of Dido in human carcinoma cells compared to normal non-cancerous cells, PNT2 and ARPE19 ​cells.

Carcinoma cell lines	Compared to PNT2	Compared to ARPE119
PC3	2.99	4.8
DU145	3.57	5.73
A2780	2.47	3.97
A2780-CP70	2.06	3.32

**Fig. 3:**
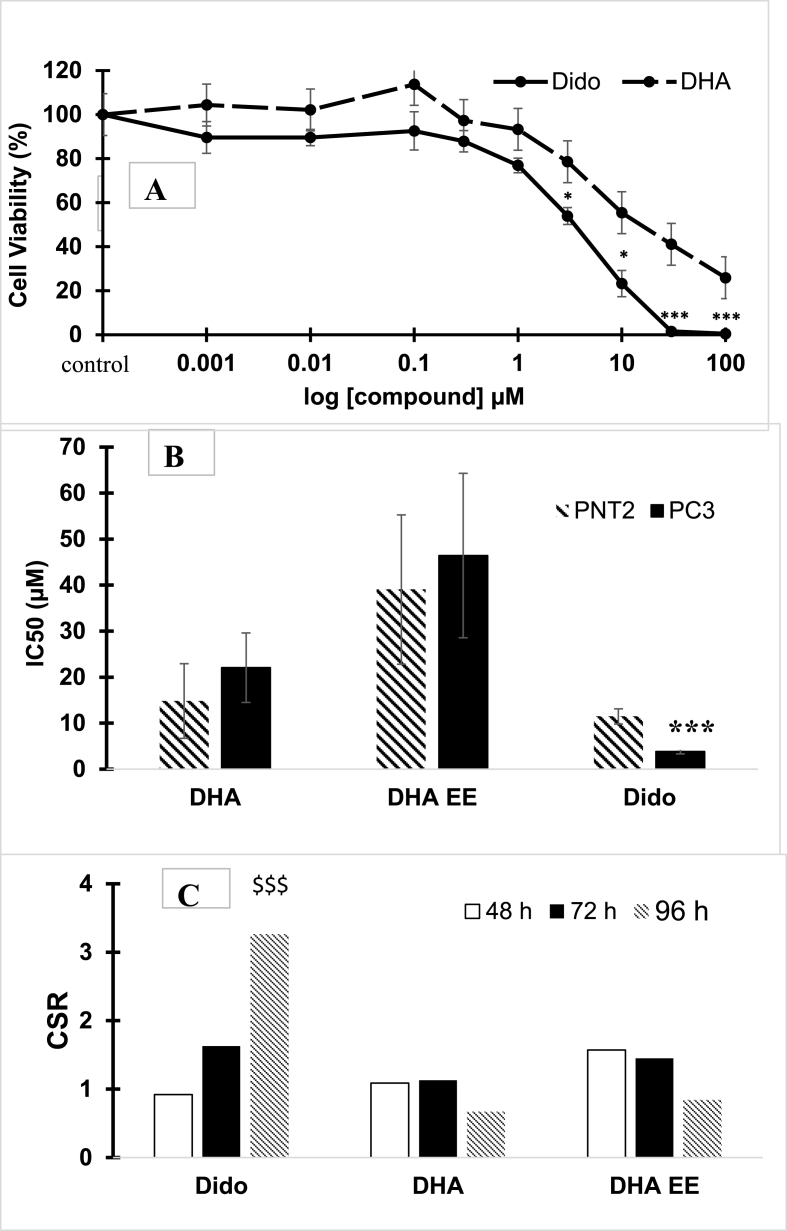
The effect of Dido and DHA over 96 ​h exposure to (A) human prostate cancer cell line PC3, and (B) Comparison of IC_50_ values of DHA, ethyl docosahexaenoate (DHA EE) and Dido following 96 ​h exposure of PC3 prostate cancer cell line compared to normal human prostate cancer cell line PNT2. (C) Comparison of cytotoxicity between PNT2 and PC3 cells following 48, 72 and 96 ​h exposure to Dido, DHA and DHA EE. Each point represents mean ​± ​s.e.m of n ​= ​4, ∗p ​< ​0.05, ∗∗∗p ​< ​0.001 were taken significant compared to control, $$$ p ​< ​0.001 compared to other contact times, DHA and DHA EEE.

In further experiments the cytotoxicity of Dido was compared with that generated by DHA (IC50 of 22.05 ​μM) and DHA EE (IC50 of 39.03 ​μM). Dido was 5.76 and 12.15-fold more cytotoxic to PC3 cells than was treatment with DHA and DHA EE, respectively and 3.26-fold more selective (p ​< ​0.01) when compared to effects in normal prostate cells (PNT2) following a 96 ​h exposure time (Fig. 3). Not only was Dido more potent on PC3 cells than PNT2 when compared to both DHA and DHA EE, but it was also selective, being more toxic towards PC3 cells in a time dependent manner (Fig. 3). Therefore, subsequent experiments investigating the mechanism of action of Dido were carried out on human prostate carcinoma cell lines, PC3 cells.

### 3.2 Investigation of cell apoptosis induced by Dido in PC3 cells

#### 3.2.1 Annexin assay and caspase 3/7 activation

**Fig. 4:**
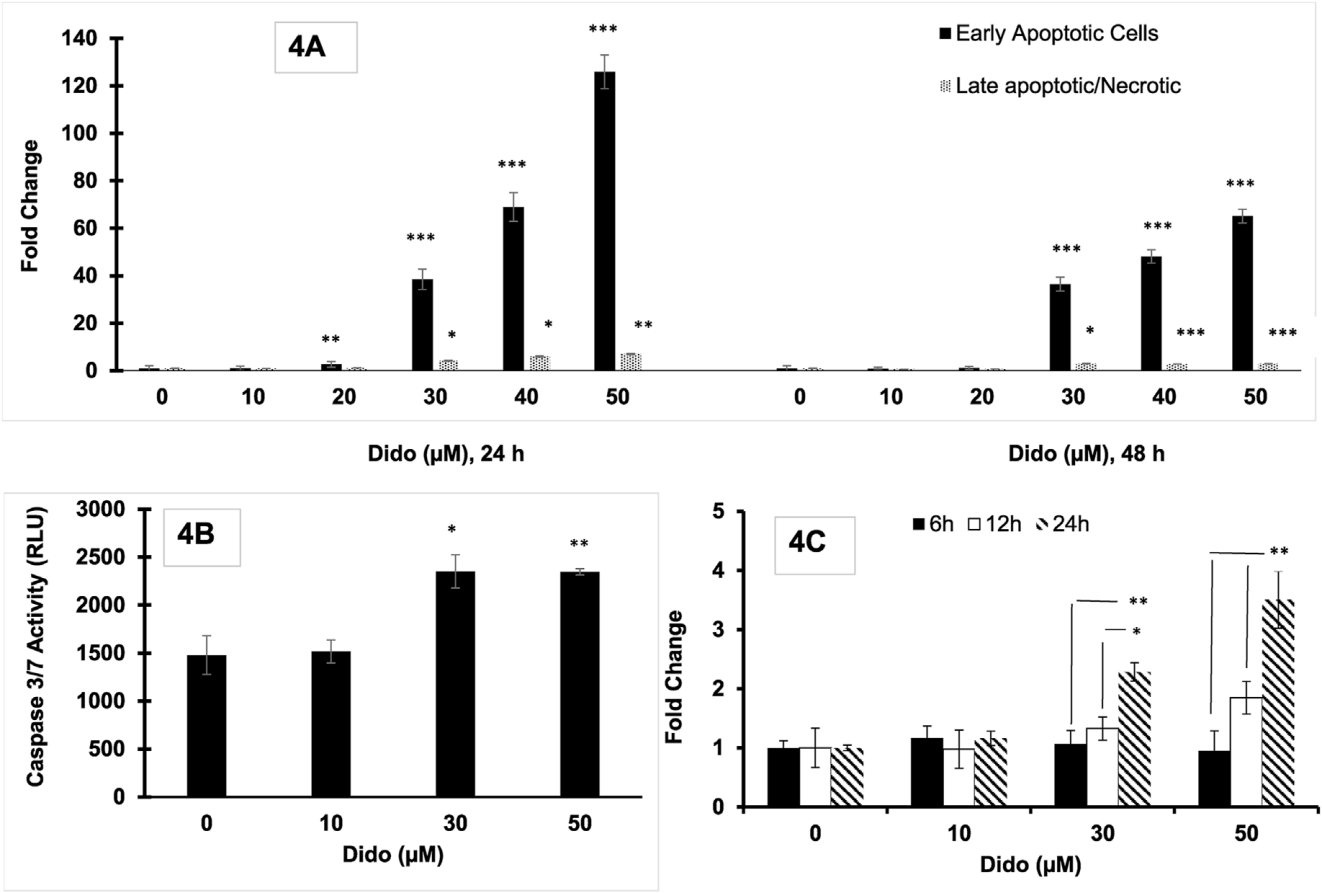
(A) Annexin assay: Fold change of PC3 cells treated with Dido, 10, 20, 30, 40 and 50 ​μM or vehicle (1% v/v) over 24- and 48-h exposure normalised with basal level expression using PI/Annexin V staining. (B) Caspase 3/7 activity of Dido (10, 30 and 50 ​μM) or vehicle treated PC3 human prostate carcinoma cells following 6 ​h exposure. (C) Mitochondrial Membrane Potential assay: Fold change of PC3 cells treated with Dido (10, 30 and 50 ​μM) or vehicle (1% v/v) over 6-, 12- and 24-h exposure normalised with basal level expression using JC-1 staining. Data is expressed as mean ​± ​s.e.m, n ​= ​3, statistical significance expressed as ∗ p ​< ​0.05, ∗∗p ​< ​0.01, ∗∗∗p ​< ​0.001 compared to control. Data is expressed as mean ​± ​s.e.m, n ​= ​3, statistical significance expressed as ∗ p ​< ​0.05, ∗∗p ​< ​0.01, ∗∗∗p ​< ​0.001 compared to control.

The data strongly suggest an induction of apoptotic cell death after 24 and 48 ​h with relatively increased quantities of early apoptotic cells due to the externalisation of phosphotidyl serine from exposure to Dido as can be seen in Fig. 4A. Fold changes of early apoptotic cells increased incrementally after 24 ​h exposure with fold increases of 1.04, 2.64 (p ​< ​0.01), 38.5 (p ​< ​0.001) and 68.9, 125.9 (p ​< ​0.001) for Dido concentrations of 10, 20, 30, 40 and 50 ​μM, respectively. Elevated levels of early apoptotic cells were also observed following 48 ​h exposure, with a 36.5 (p ​< ​0.001) fold increase with Dido treated PC3 cells rising to 48.1 and 65.1 (p ​< ​0.001) when used at a concentration of 40 and 50 ​μM, respectively relative to the baseline control at the same timepoint (Fig. 4A).

Treatment of PC3 cells with Dido for 6 ​h at 30 and 50 ​μM significantly (p ​< ​0.05; p ​< ​0.01) increased the level of activated caspase 3/7 (Fig. 4B).

#### 3.2.2 Mitochondrial membrane potential assay and investigation of ROS formation induced by Dido in PC3 cells

Following 6 ​h exposure time to Dido, there was no noticeable fold change in the membrane potential compared to the vehicle treated cells. However, when cells were exposed to Dido at 30 and 50 ​μM for 24 ​h contact time there was a significant fold change increase (p ​< ​0.05, p ​< ​0.01) in the loss of mitochondrial membrane potential (Fig. 4C).

Pre-treatment with the antioxidants NAC (N-acetylcysteine), ascorbic acid (vitamin C) and α-tocopherol (vitamin E) induced a protective effect against Dido (100 ​nM–30 ​μM) -induced cytotoxicity in the PC3 cancer cell line and PNT2 cells (Table 2). The data showed an IC_50_ of 21.79 ​± ​2.21 ​μM for Dido alone when it was in contact with the cells for 24 ​h, which did not reach the same level of cytotoxicity when Dido was added to the cells in the presence of any of the three antioxidants used. In other words, Dido was much less cytotoxic to the cells in the presence of antioxidants and consequentially the IC_50_s could not be calculated. When Dido was in contact with the cells in the presence of any of the antioxidants for 96 ​h the IC_50_s were either not calculatable or were significantly (p ​< ​0.001) greater than IC_50_s in the absence of the antioxidants (Table 2). Interestingly, α-tocopherol was shown to have the greatest protective effect on Dido-induced cytotoxicity since the cytotoxicity did not reach 50%.

**Table 2:** The IC50 (μM) values for Dido in the absence and presence of different antioxidants in human prostate carcinoma (PC3) and normal cells (PNT2). ∗∗∗p ​< ​0.001 compared to the effect of Dido alone.

Drug/Antioxidant combination	PC3 24 ​h	PNT2 24 ​h	PC3 96 ​h	PNT2 96 ​h
Dido	21.79 ​± ​2.21	18.53 ​± ​1.03	3.82 ​± ​0.51	11.46 ​± ​1.65
Ascorbic acid (250 ​μM) ​+ ​Dido	>50	>50	22.12 ​± ​5.00 ∗∗∗	14.81 ​± ​1.90
NAC (500 ​μM) ​+ ​Dido	>50	41.82 ​± ​3.01 ∗∗∗	>50	12.89 ​± ​2.81
α-tocopherol (5 ​μM) ​+ ​Dido	>50	>50	>50	>50

**Fig. 5:**
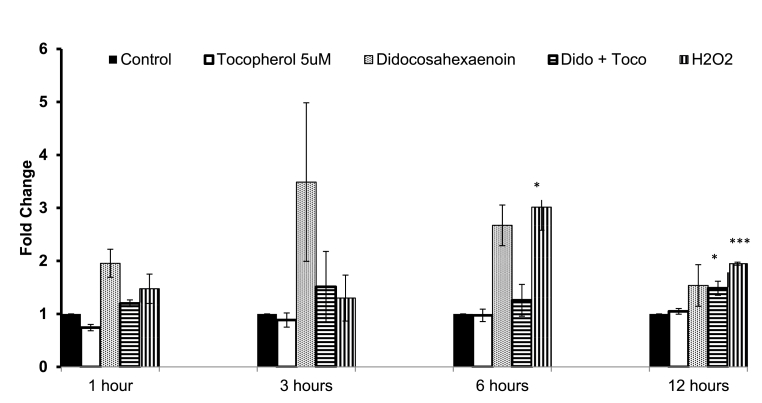
Fold change of presence of fluorescent 2,7-dichlorohydrofluorescein after exposure to α-tocopherol (5 ​μM), didocosahexaenoin (Dido, 30 ​μM), a combination of Dido (30 ​μM) plus α-tocopherol (5 ​μM), hydrogen peroxide (50 ​μM) or ethanol over 1, 3, 6 and 12 ​h in PC3 human prostate carcinoma cells. Data is expressed as mean ​± ​s.e.m, n ​= ​3, statistical significance is expressed as ∗ p ​< ​0.05, ∗∗∗p ​< ​0.001 for Dido compared to vehicle control.

Further experiments were carried out where the level of 2′7′-dichlorodihydrofluorescein fluorescence was assessed in cells treated with Dido at 30 ​μM in the absence and presence of α-tocopherol at 5 ​μM. Results showed that Dido at 30 ​μM induced 2′7′-dichlorodihydrofluorescein fluorescence 2-fold greater than that of basal expression after 1 ​h exposure, over 3-fold greater after 3 ​h and 2.8-fold after 6 ​h exposure time. Pre-treatment with α-tocopherol (5 ​μM) plus Dido (30 ​μM) induced a reduction in 2′7′-dichlorodihydrofluorescein fluorescence at all the time points tested, with greatest effects seen at 3 and 6 ​h contact times. Hydrogen peroxide (50 ​μM) was used as a positive control and was most effective at 6 ​h. Pre-treatment of cells with α-tocopherol (5 ​μM) alone induced similar 2′7′-dichlorodihydrofluorescein fluorescence to that of the vehicle-treated control cells (Fig. 5).

## 4 Discussion

The present study is the first to report potent anti-tumour activity induced by Didocosahexaenoin (Dido), a DHA diglyceride derivative, when tested on human prostate carcinoma cells, PC3 and DU145 ​cells. The studies showed IC_50_ values of less than 5 ​μM. This prompted a further testing on other carcinoma cells such as ovarian and breast carcinoma cells in order to establish whether the cytotoxicity induced by Dido is specific to prostate carcinoma cells. Interestingly Dido was able to induce cytotoxicity in these cell lines, however with lower potency. Another interesting observation was that Dido was much more potent as a cytotoxic agent than DHA or DHA EE when tested on prostate carcinoma cells. PC3 and DU145 were selected as representatives of aggressive prostate cancers, to which Dido showed a cytotoxicity selectivity ratio of 3.26 (p ​< ​0.01) and 3.88 (p ​< ​0.01) for PC3 and DU145, respectively when compared with PNT2, normal/non-cancerous prostate cells.

In an attempt to investigate the involvement of intrinsic pathways in mediating the cytotoxicityinduced by Dido, the present study showed that Dido induced ROS production. This observation was in line with previous studies which used DHA and reported cytotoxicity and ROS production in apoptotic cell death at sub 100 ​μM concentrations (Shin et al., 2013; Kang et al., 2010; Jing et al., 2011). The significance of ROS production in Dido-induced cell death in prostate carcinoma cells is also supported by the observations made in the present study when pre-treatment with various antioxidants significantly protected or delayed the cytotoxicity. Previous studies have shown a direct relationship between an overexpression of Bach1 and increase in ROS activity in mitochondria (Wang et al., 2016). Our results indicated an increase in the level of ROS leading to apoptosis which might be the result of an increase in the level of Bach1. The attenuation of the cytotoxicity-induced by Dido when cells were pre-treated with NAC (a ROS scavenger), further suggests the likely involvement of Bach1 in mediating apoptosis. This is in line with the previous studies that also indicated an increase in the level of Bach1 which led to an increase in the level of ROS-induced apoptosis which was also sensitive to NAC treatment (Wang et al., 2016). Therefore, it is possible that the induction of apoptosis is through an intrinsic pathway which in turn is regulated by a balance between the proapoptotic and antiapoptotic Bcl2 proteins in mitochondria (Coultas and Strasser, 2003; Green and Kroemer, 2004). Thus, further experiments are required to substantiate the involvement of Bach1 in mediating the cytotoxicity-induced by Dido. However, this does not negate the possibility that ROS are released as a side effect of the activation of another pathway. For example, it has been shown that activation of caspases- 3 and -9 resulted in ROS production as a side effect which was not related to the key signalling events which lead to the caspase-dependent cell death (Cai and Jones, 1998). Interestingly the present study also indicated an involvement of caspases in the induction of apoptosis, which might lead to the conclusion that ROS might have a secondary role in mediating apoptosis or might be a side effect of an activation of caspases-induced apoptosis. Thus, whether ROS production is a cause of apoptosis, or a symptom of early apoptosis, might relate to the amount/actual level of ROS release and this in the current study cannot be determined.

Our studies not only confirmed an involvement for ROS pathway but also a caspase 3/7-dependent pathway in the induction of apoptosis. This was in line with previous studies where involvement of caspases and ROS were also reported in mediating the cytotoxicity afforded by the omega-3 fatty acids, such as DHA and EPA on prostate cancer (So et al., 2015; Shin et al., 2013; Kang et al.,2010; Jing et al., 2011; Wang et al., 2016). DHA has been shown to initiate both caspase 8 and 9, leading to the induction of caspase 3/7 which in turn leads to apoptosis (Shin et al., 2013). Therefore, it can be suggested that an increase in the level of ROS following treatment with Dido leads to the activation of intrinsic pathway by an increase in the level of cleaved caspase 3/7 which in turn leads to apoptosis. However, as indicated earlier it is also possible that the activation of caspases happens prior to the increase in the level of ROS. The activation of the extrinsic pathway by Dido through an activation of caspase-8 and caspase -10 remains to be investigated.

In further studies, attempts were made to examine whether Dido inhibited the growth of PC3 cells by means of affecting the mitochondrial membrane potential. The mitochondrial dysfunction due to change in membrane potential is one of the major characteristics of the activation of intrinsic pathway leading to apoptosis (Goc et al., 2012). The present study showed that pre-treatment with Dido increased mitochondrial membrane potential. This finding is in line with previous studies which showed the induction of apoptosis through change in the mitochondrial membrane potential in neuroblastoma cells treated with omega-3 fatty acids such as DHA and EPA (So et al., 2015).

Further and final evidence for the induction of apoptosis by Dido came from experiments using annexin-V/PI. Such experiments showed that pre-treatment with Dido increased quantities of early apoptotic cells. This was in line with previous studies where other omega-3 fatty acids such as DHA and EPA induced apoptosis by increasing the externalisation of PS (So et al., 2015). Externalisation of Phosphatidylserine (PS), which in normal cells resides mainly in the inner leaflet of the plasma membrane, is one of the most prominent features of cell death. In apoptotic cells specific enzymes, scramblases are involved in translocating the PS from the inner leaflet of plasma membrane to the outer leaflet where Annexin V can be conjugated. It has been shown that activation of caspases- 3 and -7 are able to activate lipid scramblase which in turn leads to PS exposure in the outer leaflets of plasma membrane or extracellular space (Marino and Kroemer, 2013).

Finally, it was an interesting observation that Dido could also induce strong cytotoxicity in other cell lines such as ovarian and breast carcinoma cells and notably resistant carcinoma cells such as A2780-CP70. Previous studies showed that DHA improved tumour cytotoxicity of drugs in P388 and P388/DOX (doxorubicin resistant) mouse leukaemia cells by improving the uptake of Doxorubicin/*cis*-platin (Merendino et al., 2013). However, the same study showed that DHA failed to improve the cytotoxicity of drugs against human MDA-MB-231 and MCF-7 doxorubicin resistant cells. It remains to be investigated if the sensitivity of resistant cells to cytotoxicity afforded by Dido would also involve an improved uptake of chemotherapeutic drugs.

In summary, the present study is the first to provide evidence for Dido to induce a much more potent cytotoxicity than DHA, with a high preference towards PC3 carcinoma cells over normal non-carcinoma cells. Our studies also suggest an induction of apoptosis which may involve an activation of ROS pathway, a caspase-3/7 dependent pathway as well as a loss in the mitochondrial membrane potential. Activation of intrinsic pathway is likely to be due to the mitochondrial dysfunction, externalisation of phosphatidyl serine and possibly an increase in the caspase-3/7 activity.

Although the ability of Dido to simultaneously modulate and target the intrinsic and extrinsic pathways in mediating apoptosis would create a complexity in defining a specific mechanism, this however might be beneficial in chemo-resistant tumours. Carcinoma cells are known to develop resistance against treatments targeting specific pathways, the use of omega-3 fatty acids such as Dido may offer the benefit of multi-pathway activation in counteracting the acquired resistance mechanism. It will also be interesting to test whether pre-treatment with Dido can increase the sensitivity of carcinoma cells to the standard chemotherapeutic drugs and radiotherapy. This necessitates further preclinical and clinical research into the use of omega-3 fatty acids and in particular Dido in cancer treatment. Dido might have a potential as an anti-cancer agent which might be used for the adjuvant therapy or combination therapy with conventional chemotherapeutic drugs.

## CRediT authorship contribution statement

**Glenn F. Robinson:** Writing – original draft, Writing – review & editing. **Roger M. Phillips:** Writing – review & editing. **Simon J. Allison:** Supervision. **Farideh A. Javid:** Supervision, Writing – review & editing.

## Declaration of competing interest

The authors declare the following financial interests/personal relationships which may be considered as potential competing interests: Dr Glenn Robinson reports financial support was provided by Dr Brian Whittle Ltds.
